# A novel bacteriophage KSL-1 of 2-Keto-gluconic acid producer *Pseudomonas fluorescens* K1005: isolation, characterization and its remedial action

**DOI:** 10.1186/1471-2180-12-127

**Published:** 2012-06-29

**Authors:** Wen-Jing Sun, Chang-Feng Liu, Lin Yu, Feng-Jie Cui, Qiang Zhou, Si-Lian Yu, Lei Sun

**Affiliations:** 1 School of Food and Biological Engineering, Jiangsu University, Xuefu Rd., Zhenjiang City, Jiangsu Province, 212013, People's Republic of China; 2 Parchn Sodium Isovitamin C Co. Ltd, Xingangshan Country, Dexing City, Jiangxi Province, 334221, People's Republic of China; 3 Laboratory of Pharmaceutical Engineering, School of Medicine and Pharmaceutics, Jiangnan University, No.1 Weigang, Wuxi, 214122, People's Republic of China

## Abstract

**Background:**

Bacteriophages have the destructive damage on the industrial bioprocess. 2-Keto-gluconic acid (2KGA) producing bacteria had also been attacked and lysed by bacteriophages which lowered the glucose consumption and 2KGA yield and even stopped the fermentation process. In this study, we presented the characteristics of a novel virulent bacteriophage specifically infecting *Pseudomonas fluorescens* K1005 and proposed an efficient remedial action for this phage infection to reduce the production loss.

**Results:**

The phage KSL-1 of *Pseudomonas fluorescens* K1005 was isolated from abnormal 2KGA fermentation broth. It belonged to the Siphoviridae family with a hexagonal head diameter of about 99 nm and a non-contractile tail of about 103 nm × 39 nm. The genome size of phage KSL-1 was estimated to be approximately 53 kbp. Its optimal MOI to infect *P. fluorescens* K1005 was about 0.001. One-step growth curve gave its latent and burst periods of 90 min and 75 min with a burst size of 52 phage particles per infected cell. This phage was stable with a pH range of 7.0–10.0, and sensitive to thermal treatment. Finally, a simple remedial action was proposed by feeding fresh seed culture. Compared with the infected 2KGA fermentation, the remedial experiments restored 2KGA fermentation performance by increasing the produced 2KGA concentration to 159.89 g/L and shortening the total fermentation time of 80 h with the productivity and yield of 2.0 g/L.h and 0.89 g/g. The obtained data proved that this method was effective to combat the phage infections problems during the 2KGA fermentation.

**Conclusion:**

The phage KSL-1 was a novel bacteriophage specifically infecting *Pseudomonas fluorescens* K1005. The remedial action of feeding fresh seed culture to the infected broth was an easily-operating and effective method to maintain a high 2KGA yield and avoid the draft of infected broth.

## Background

Modern industrial-scale fermentations increasingly rely on the cultivated bacteria to drive product formation. However, bacteriophages (phages) have the potential to directly interfere with any fermentation industry by attacking and lysing the industrial bacteria [1-3]. The industrial decontamination of bacteriophage infection may be more complex comparing with laboratory scale since a phage propagated in a bioreactor can spread throughout the plant leading to a wide spread of phage, complete loss of the desired bioproduct, and significantly economic reduction of plants. For example, Acetone Butanol (AB) solvent yield at the plant had been cut by half for almost a year due to the presence of phages in bioprocessing environments [4]. Although the deleterious effect caused by bacteriophages was known to those working with bacteria, there are relatively few published reports addressing this problem and finding descriptions in industrial bioprocesses [4].

Some procedures may prevent phage infection of bacterial cultures. Good laboratory/factory hygiene, sterilization, decontamination, and disinfection are absolutely necessary to avoid fatal events caused by bacteriophages. However, all these procedures cannot guarantee the absence of phage contamination [5]. When a phage infection did occur, the standard practice was to eliminate all of the contaminated material, followed by cleaning and sterilization. The infected broth in tons will be drafted in an industrial case which led to the direct cost loss and environmental problems. Hence, to seek an economic treatment procedure or remedial method is a definite interest for industrial plants.

2-keto-d-gluconic acid (2KGA) is a key organic acid due to its intermediate role in the manufacture of erythorbic acid, an antioxidant widely used in food industry [6]. It is produced in an industrial scale by various bacteria including *Cluconobacter oxydansPseudogluconobacterPseudogluconobacter saccharoketogenes*, and *Pseudomonas sorbosoxida*[6-9]. Similarly, bacteriophages attack and lyse the 2KGA producing bacteria to lower substrate consumption or end-product yield and even stop the fermentation process. For example, a serious bacteriophage infection of 2KGA fermentation occurred widely in most Chinese plants in spring of 1999 [9]. Five bacteriophages (KS502, KS503, KS211, KS212 and KS213) had been isolated from the abnormal *Pseudomonas fluorescens* K1005 and *Arthrobacter globiformis* K1022 cultured broth [10,11]. The new immunized strains including *P. fluorescens* AR3, AR4, AR12 and AR16 were generated to counter the phage contamination [12]. However, the repercussions caused by the phage infections still reoccurred in majority of Chinese 2KGA producing factories. Thus, besides scrupulous hygiene and screening immunised strains, the characteristic knowledge of bacterial phages and the economical remedial treatments were still needed for 2KGA industrial factories.

This present study will focus on: 1) isolating and characterizing of a novel phage specifically infecting *Pseudomonas fluorescens* K1005 in the abnormal 2KGA industrial fermentation, and 2) proposing an effective and economical remedial action to complete the production process with high 2KGA fermentation performance.

## Results and discussion

### Isolation and morphology of bacteriophage KSL-1

Abnormal fermentation broth samples from a 2KGA production plant were used to detect the presence of phages against the indicator strain of *Ps. fluorescens* K1005. Only one type of phage was isolated, purified and designated as KSL-1. It showed the lytic activity and high specificity towards its host bacteria *Pseudomonas fluorescens* K1005. Other tested *Pseudomonas fluorescens* strains of A46 and AR4 could not be infected by the phage KSL-1.

**Figure 1:**
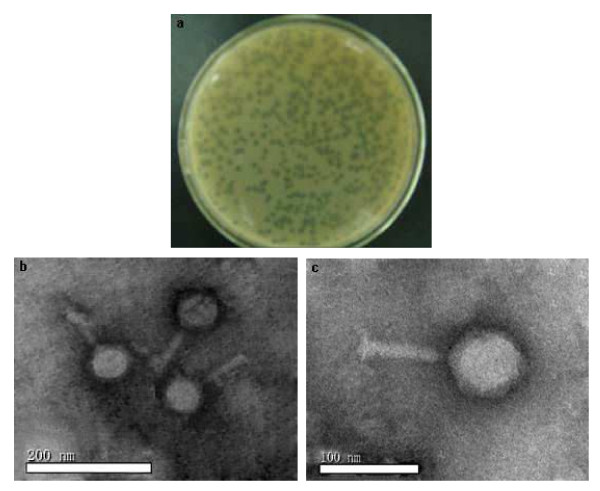
**Morphology of phage KSL-1. a) Plaques of phage KSL-1; b) and c) electron micrograph of phage KSL-1, phage KSL-1 were negatively stained with 2% (w/v) phosphotungstic acid.** Magnification: 37,000 × and 135000×, respectively.

The phage KSL-1 formed small, round plaques (about 1.0 mm in diameter) with transparent middle and turbid edge slightly on the double-layer plate (Figure 1a). The electron micrographs (Figure 1b and c) showed that KSL-1 has a hexagonal head diameter of about 99 nm and a non-contractile tail of about 103 nm × 39 nm. According to the International Committee on Taxonomy of Viruses, the phage KSL-1 belonged to family Siphoviridae [13,14].

### DNA characterization

**Figure 2:**
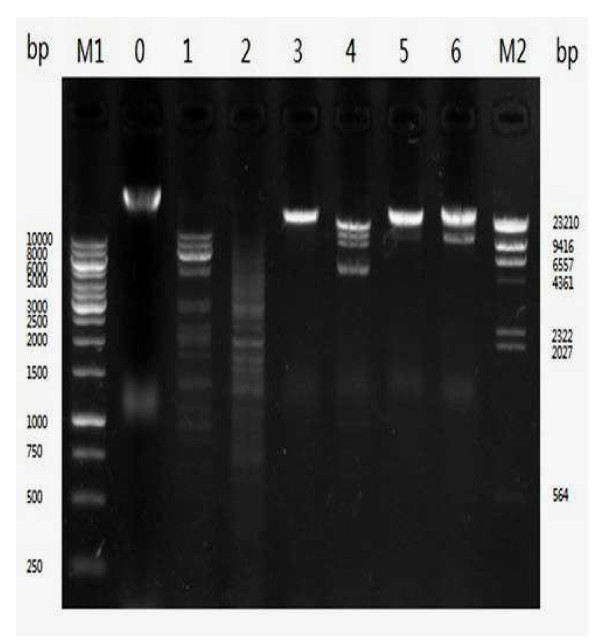
**Agarose gel electrophoresis showing restriction fragments generated from digesting phage KSL-1 DNA with endonuclease.** Lanes are as follows: M1,Takara λHind III DNA Marker; M2, GeneRuler 1Kb DNA Ladder; 0, undigested; 1, EcoR I; 2, Hind III; 3, BamH I; 4, SnaB I; 5, Sal I; 6, Sac I.

The restriction patterns of phage KSL-1 (Figure 2) were obtained with restriction endonucleases (EcoR I, Hind III, BamH I, SnaB I, Sal I and Sac I). Like most tailed phage, the genome was found to be double-stranded DNA. The genome size was determined to be approximately 53 kb (lane 4) running it with λHind III DNA marker and GeneRuler 1Kb DNA ladder on 0.8% agarose gel, which was different from *Pseudomonas fluorescens* phage φIBB-PF7A(42 kb) [15]. Although the genome size of the phage KSL-1 was similar to phage ΦGP100 (50 kb), the morphologies of these two phages had significant difference [16].

### Optimal multiplicity of infection (MOI) of KSL-1

**Figure 3:**
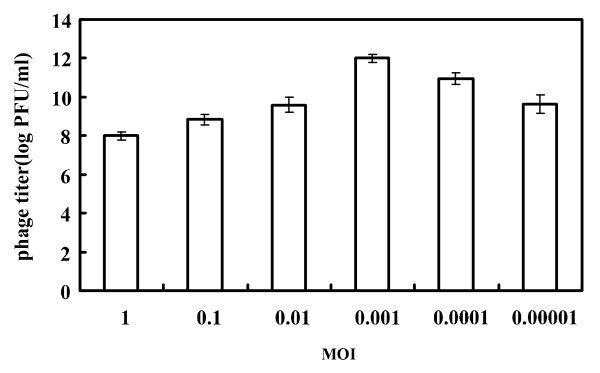
**Optimal multiplicity of infection (MOI) of phage KSL-1.** Comparison of phage titer after incubation for 3.5 h at six ratios of MIO (0.00001, 0.0001, 0.001, 0.01, 0.1 and 1 PFU/CFU) in LB medium.

The MOI resulting in the highest phage titer was considered to be optimal for the following experiments [17]. In the present study, the optimal MOI of phage KSL-1 was determined to be 0.001, i.e., KSL-1 lysate of about 10 × 10^11^/mL would be obtained (Figure 3).

### One-step growth curve

**Figure 4:**
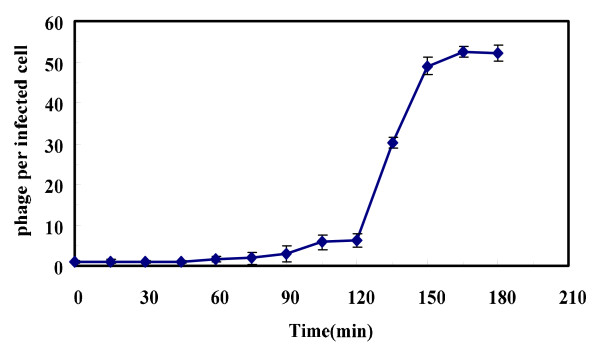
One-step growth curve of phage KSL-1.

The one-step growth curve experiment of KSL-1 was performed for determining the latent time period and burst size of phage. There is a progressive relationship between burst size and latent period such that an optimal latent period leads to high phage fitness, an upsurge in burst size may contribute to plaque size or larger plaques with higher burst size [18,19]. Burst size is calculated as the ratio of the final count of liberated phage particles to the initial count of infected bacterial cells during the latent period [20]. Burst size and latent period are influenced by host, medium compositions and incubation temperature and specific growth rate [21]. From Figure 4, the latent period was calculated to be 90 min. the burst time was 75 min and the calculated burst size was about 52 phage particles per infected cell.

### Factors affecting phage KSL-1 stability

**Figure 5:**
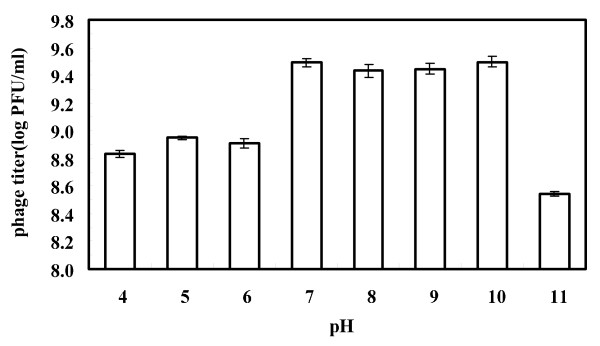
**Effect of pH on phage KSL-1 stability.** Phage was incubated under different pH values for 60 min in 1.0% peptone solution at 25 ±0.3°C.

As shown in Figure 5, after 60 min incubation the phage titers decreased from the initial incubated level of 9.5 log PFU/mL to about 8.8 log PFU/mL, 8.9 log PFU/mL and 8.9 log PFU/mL at pH 4.0, 5.0 and 6.0, respectively, while a sharp decrease appeared to be about 8.5 log PFU/ml when pH value was set as 11.0. Scarcely any reduction of the phage titer was observed at other pH values (7.0, 8.0, 9.0 and 10.0). The obtained results showed that the phage KSL-1 was stable at wide alkaline pH range.

**Figure 6:**
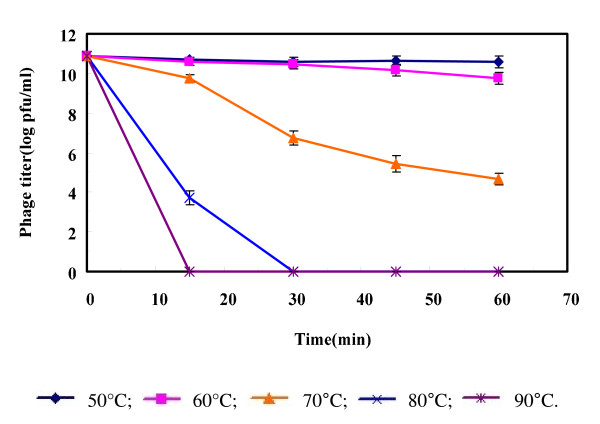
**Inactivation kinetics of phage KSL-1 at different temperature**.

Thermal stability tests were carried out to analyze the heat-resistant capability of phage KSL-1 at 50°C, 60°C, 70°C, 80°C and 90°C. Survivor curves of the phage KSL-1 are shown in Figure 6. After 60 min of thermal treatment, the phage retained almost 100% survivor at 50°C. The reduction was calculated as only 1.1 log at 60°C and 6.2 log at 70°C. The phage survivor was reduced by 7.1 log after 15 min at 80°C. No phages were remained at 80°C after 30 min or at 90°C after 15 min. Therefore, phage KSL-1 showed the sensitivity to thermal treatment with temperature of over 80°C. These obtained data would also provide a reference for taking control of the serious phage infection consequences by using boiling water to rinse all heat resistant equipment and to clean working areas [1,3].

### Effect of phage KSL-1 on the 2KGA production

**Figure 7:**
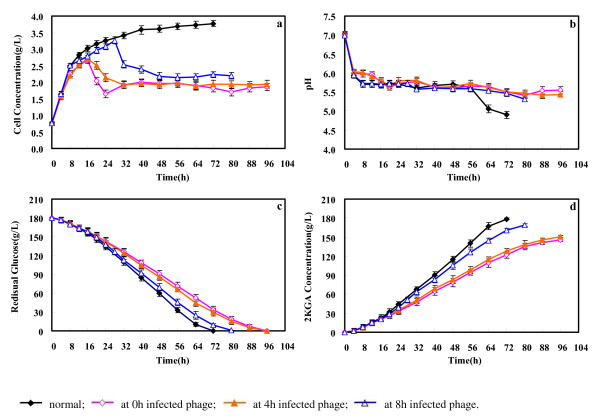
**Effect of phage infection at different stages on 2KGA production performance of*Pseudomonas fluorescens*k1005**.

Figure 7 compared the fermentation characteristics of strain *Ps. fluorescens* K1005 without or with the infection of phage KSL-1 when cultured for 0, 4 and 8 h. The normal fermentation process (without phage KSL-1 infection) showed the typical bacterial growth curve. Cell concentration increased rapidly to 2.50 g/L in the earlier 8 h and ended up to 3.77 g/L. pH value decreased from 7.02 and kept the stable level of 4.90 with the balance of CaCO_3_. The produced 2KGA concentration was 178.45 g/L from 180 g/L of glucose after 72-h fermentation. The final productivity was 2.48 g/L.h with a yield of 0.99 g/g.

Phage infections affected the bacterial growth and 2KGA production performance. When infected with KSL-1 at 0^th^ hour, the total fermentation time prolonged to 96 h. Cell concentration increased slowly to 2.67 g/L after 16-h cultivation, and decreased to 1.86 g/L at the end of fermentation. About 144.98 g/L of 2KGA was produced. Compared to normal fermentation, productivity and yield decreased to 1.51 g g/L.h and 0.81 g/g, respectively. The fermentation performance presented similar pattern when infected with KSL-1 at 4^th^ hour. However, the phage infection at 8^th^ h of fermentation had the difference with other two experiments. The fermentation time shortened to 80 h, cell concentration began to decrease from 3.26 g/L after 28-h cultivation to the final level of 2.20 g/L, and final productivity and yield were 2.11 g/L.h and 0.94 g/g, respectively. The burst time and size of phage and host cell concentration possibly co-contributed to this difference.

### Feeding seed culture to the infected fermentation broth as efficient remedial action

**Figure 8:**
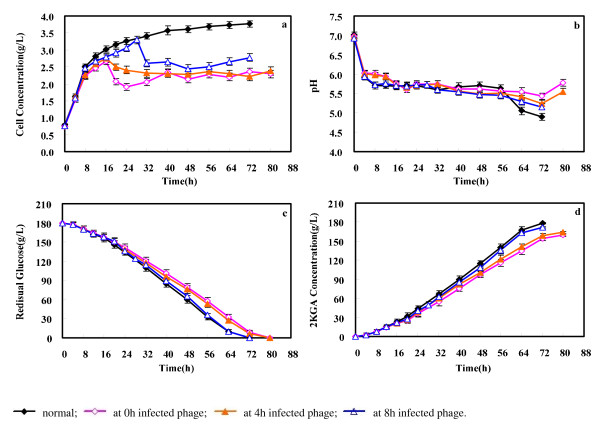
**Effect of feeding seed cuture for phage infection in the 2-Keto-Gluconic Acid (2KGA) fermentation process**.

Sterile conditions and resistant strains to phages are the successful and widespread approaches, but could not prevent a phage infection. The phage-infected fermentation broth had to be discharged after chemical treatment, and no effective means of salvaging phage-contaminated fermentation broths were ever developed. Herein, feeding seed culture to the fermentation broth was proposed as an effective remedial action and shown in Figure 8.

As for the infection of phage KSL-1 at 0^th^ hour, when cell concentration decreased to 2.07 g/L at the 20 h of fermentation, fresh seed culture was fed. 2KGA fermentation continued to the endpoint with the produced 2KGA concentration of 159.89 g/L, which was 1.11 times of that infected fermentation at 0^th^ hour without seed culture feeding. The total fermentation time decreased to 80 h with the complete consumption of glucose, and the productivity and yield of 2KGA increased to 2.0 g/L.h and 0.89 g/g. Interestingly, cell concentration showed a waving model which may contribute to the bacterial succession and co-evolution of bacteria and their viruses in an arms race [22].

When feeding fresh seed culture into the 8^th^ -h infected fermentation broth, fermentation time decreased to 72 h which comparable to the normal process. 2KGA concentration increased slightly from 168.85 g/L to 171.34 g/L. Table 1 summarized the overall fermentation performances of 2KGA production under the conditions of normal and phage infection with/without feeding fresh seed culture at various infection stages. Therefore, feeding fresh seed culture to infected fermentation broth was proposed once the cell concentration began to decrease after phage infection. And this proposed remedial action was effective to obtain the desirable 2KGA fermentation performance without stopping the 2KGA production process and discharging the infected broth.

**Table 1:** **Summary of 2KGA production from phage infection at different stages by*Pseudomonas fluorescens*K1005**

**Parameters**		**Without feeding seed cuture**			**With feeding seed cuture**		
	**Normal**	**Infected phage at 0 h**	**Infected phage at 4 h**	**Infected phage at 8 h**	**Infected phage at 0 h**	**Infected phage at 4 h**	**Infected phage at 8 h**
Fermentation periods (h)	72	96	96	80	80	80	72
2KGA concentration (g/L)	178.45 ± 1.41	144.98 ± 1.61	150.79 ± 1.42	168.85 ± 1.95	159.89 ± 2.52	163.59 ± 1.55	171.34 ± 1.25
percent conversion(%)	91.99 ± 0.71	74.73 ± 0.83	77.73 ± 0.74	87.04 ± 1.00	82.42 ± 1.30	84.32 ± 0.80	88.32 ± 0.64
Total productivity (g/L.h)	2.48 ± 0.02	1.51 ± 0.01	1.57 ± 0.01	2.11 ± 0.03	2.00 ± 0.30	2.04 ± 0.02	2.38 ± 0.01
Maximum productivity (g/L.h)	2.61 ± 0.13	1.71 ± 0.17	1.79 ± 0.04	2.26 ± 0.05	2.15 ± 0.17	2.21 ± 0.06	2.54 ± 0.04
Yield (g/g)	0.99 ± 0.01	0.81 ± 0.01	0.84 ± 0.01	0.94 ± 0.01	0.89 ± 0.01	0.91 ± 0.01	0.95 ± 0.01

## Conclusions

The isolation and characterization of a specifically-infecting phage KSL-1 to 2KGA producer *Ps. fluorescens* K1005 provided valuable information including its morphology, molecular structure and physical stability. This phage significantly affected bacterial growth and 2KGA production performance. To avoid stopping 2KGA production process, discharging the infected fermentation broth, and saving the cost of production process, a remedial action with feeding fresh seed culture was proposed and proven to be an easily-operating and effective method. Further scale-up experimentation is ongoing in the collaborative company and our lab.

## Materials and methods

### Bacterial strain, bacteriophages and culture media

*Ps. fluorescens* K1005 was screened and kept in our laboratory [10] and used as a sensitive strain. The bacterial stock cultures were stored at −4°C in agar slant containing peptone 10.0 g/L, beef extract 5.0 g/L, NaCl 5.0 g/L and agar 20.0 g/L. The seed culture was obtained by diluting the stock culture with sterilized water, inoculating into 60 mL of seed medium containing glucose 20.0 g/L, corn steep liquor 10.0 g/L, urea 2.0 g/L, KH_2_PO_3_ 2.0 g/L, MgSO_4_·7H_2_O 0.5 g/L, CaCO_3_ 5.0 g/L, and culturing in a 500 mL Erlenmeyer flask at 30°C for 18 h. Fermentation medium consisted of glucose 180.0 g/L and corn steep liquor 20.0 g/L. CaCO_3_ 45.0 g/L was added to the medium for balancing the broth pH. Bacteriophage stocks were prepared by addition of phages to Lysogeny broth (LB) medium with an appropriate amount of *P. fluorescens* culture.

### Bacteriophage isolation, purification and propagation

Contaminated 2KGA fermentation samples were centrifuged (3500 × g for 10 min). The collected supernatant was filtered using a millipore filter (0.45 μm pore size). The double-layer plate method was used to isolate phages [18]. Well-isolated individual plaques were punctured with vaccination needle and transferred into sterile water. Plaques were purified for five times by serial dilution and plating to the double-layer plate. Final purified phages were stored at 4°C.

For bacteriophage propagation, the purified phage was inoculated to a 500 mL Erlenmeyer flask containing 50 mL of LB medium or seed medium and cultured for 24 h at 30°C with a rotatory speed of 270 rpm on rotary shaker. The obtained broth was centrifuged at 3500 × g for 10 min. The supernatant was filter-sterilized and phage enumerations (pfu/mL) were performed by the double-layer plate method.

### Electron microscopy

High titre phage stock (10^10^-10^11^ pfu/mL) was prepared as described previously. 20 μL of phage stock was placed on copper grids and natural sediment for 15 min. Phages deposited on copper grids were negatively stained with 2% (w/v) phosphotungstic acid for 30 s. The fixed phage morphology was examined with a Hitachi H-7500 transmission electron microscope.

### Phage DNA extraction

Phage DNA was extracted essentially according to the method of Sambrook et al. [23]. DNA sample was stored in TE buffer at −20°C. For restriction analyses, purified phage DNA was digested by using six endonucleases(EcoR I,Hind III,BamH I,SnaB I,Sal I and Sac I) (FastDigest™, Fermentas) according to the manufacturer’s instructions. The DNA fragments were separated by agarose (0.8%) gel electrophoresis in TAE buffer (40 mM Tris-acetate, 1 mM EDTA). The gel was stained with ethidium bromide and photographed under UV illumination.

### Determination of optimal multiplicity of infection (MOI)

Multiplicity of infection is defined as the ratio of virus particles to potential host cells [24]. The titre of prepared phage stock was determined by serial dilution and double-layer plate method. An early log phase of host strain was grown in LB medium at 30°C for 7 h and enumerated by plating samples onto LB agar and then incubated at 30°C for 24 h. Phage stock and hosts were added to LB medium according to six ratios of MIO (0.00001, 0.0001, 0.001, 0.01, 0.1 and 1 PFU/CFU). After 3.5 h of incubation at 30°C, the samples were collected for phage titer determination.

### One-step growth curve

One-step growth curves were performed as described by Leuschner et al. [25] and Pajunen et al. [26] with some modifications. Briefly, 30 mL of an early-exponential-phase culture (OD_650nm_ = 0.1–0.2) were harvested by centrifugation (10 000 × g, 5 min, 4°C) and resuspended in one-fifth of the initial volume fresh LB medium. Phages were added with an optimal MOI and allowed to adsorb for 10 min at 30°C with the rotary speed of 160 r/min. The suspension was then centrifuged at 12 000 × g for 5 min, resuspended in 30 ml of LB broth and serial dilutions of this suspension were carried out and incubated at 30°C. At regular intervals, aliquots (100 μL) of each dilution were collected for bacteriophage counts [27]. The burst time and burst size were calculated from the one-step growth curve [18].

### Factors affecting phage stability

For investigating pH sensitivity of tested phages, a modified method was used as described by Pringsulaka et al. [1]. 100 μl of phage (about 10^10^ PFU/ml) was inoculated into a 1.0% Peptone solution with a pH range (pH 4.0, 5.0, 8.0, 9.0, 10.0 and 11.0). The samples were extracted for determining the phage titer after incubating for 60 min.

Method used to determining the phage thermal stability was followed as Lu et al. [17]. A 900 μL of 1.0% Peptone solution was preheated to the designated temperature ranging from 50 to 90°C. 100 μl of phage suspension (about 10^10^ PFU/ml) was added. At regular intervals, the phage titer was determined during 60-min culture.

### 2KGA production in laboratory scale

All fermentations were carried out in 500 mL Erlenmeyer flask containing 40 mL of fermentation medium. 10% (v/v) of seed culture was inoculated and fermented for 72 h at 30°C with a rotatory speed of 270 rpm on rotary shaker.

For infected fermentations, 1 mL (10^8^ pfu/mL) of the purified phage was inoculated into the culture after 0 h, 4 h and 8 h of 2KGA fermentation. The fermentation ended until the glucose was consumed to about 0 g/L. As for the experiment of feeding seed culture to the infected 2KGA fermentation, 7.5% (v/v) of fresh seed culture was fed when the cell concentration (valued as OD_650nm_) began to decrease. The fermentation continued until the glucose was used completely. Samples were withdrawn at intervals for testing 2 KGA, residual glucose, pH and cell concentration.

### Analytical methods

Bacteriophage titer was analysed as described by Adams [18]. Briefly, 100 μl of diluted phage solution, 100 μl of a bacterial overnight culture, and 3 ml of molten agar were mixed in a glass tube and poured into a TSA containing Petri dish. Plates were incubated for 18 h before enumeration for plaque forming units (PFU).

The concentration of 2KGA was determined and calculated on the basis of glucose concentration using Polarimetry method [28]. The optical rotation degree of final sample solution was determined with WZZ-1SS Digital Automatic Polarimeter (Precision Instrument Co., Ltd., Shanghai, China). The 2KGA concentration was calculated with the standard Equation. Glucose concentration was assayed with Biosensor Analyzer (Shandong Academy of Sciences Institute of Biology, Jinan, China) at 25°C. Cell concentration was represented by optical density at 650 nm (OD_650 nm_). 2KGA production performance was evaluated based on 2KGA concentration, productivity, and yield to glucose. 2KGA productivity was defined as the amount of 2KGA produced per hour per liter. 2KGA yield was calculated by dividing the amount of 2KGA produced by the amount of glucose consumed. All fermentation tests were run in duplicate. Data analysis including analysis of variance was conducted using the SAS System (SAS Institute, Cary, NC, USA).

## Competing interests

The authors declare that they have no competing interests.

## Authors’ contributions

W-JS and F-JC conceived of the study, participated in its design and coordination, and drafted the manuscript. C-FL performed experiments and analyzed results and helped to draft the manuscript. YL, S-LY and LS performed partial experiments and analyzed results. All authors read and approved the manuscript.
